# Recent Advances in the Role of Natural Killer Cells in Acute Kidney Injury

**DOI:** 10.3389/fimmu.2020.01484

**Published:** 2020-08-04

**Authors:** Claudia Cantoni, Simona Granata, Maurizio Bruschi, Grazia Maria Spaggiari, Giovanni Candiano, Gianluigi Zaza

**Affiliations:** ^1^Laboratory of Clinical and Experimental Immunology, Integrated Department of Services and Laboratories, IRCCS Istituto Giannina Gaslini, Genoa, Italy; ^2^Department of Experimental Medicine (DIMES) and Center of Excellence for Biomedical Research (CEBR), University of Genoa, Genoa, Italy; ^3^Renal Unit, Department of Medicine, University-Hospital of Verona, Verona, Italy; ^4^Laboratory of Molecular Nephrology, IRCCS Istituto Giannina Gaslini, Genoa, Italy

**Keywords:** inflammation, natural killer (NK) cells, tubular epithelial cells, acute kidney injury, innate immunity

## Abstract

Growing evidence is revealing a central role for natural killer (NK) cells, cytotoxic cells belonging to the broad family of innate lymphoid cells (ILCs), in acute and chronic forms of renal disease. NK cell effector functions include both the recognition and elimination of virus-infected and tumor cells and the capability of sensing pathogens through Toll-like receptor (TLR) engagement. Notably, they also display immune regulatory properties, exerted thanks to their ability to secrete cytokines/chemokines and to establish interactions with different innate and adaptive immune cells. Therefore, because of their multiple functions, NK cells may have a major pathogenic role in acute kidney injury (AKI), and a better understanding of the molecular mechanisms driving NK cell activation in AKI and their downstream interactions with intrinsic renal cells and infiltrating immune cells could help to identify new potential biomarkers and to select clinically valuable novel therapeutic targets. In this review, we discuss the current literature regarding the potential involvement of NK cells in AKI.

## Introduction

Acute kidney injury (AKI) is a life-threatening multifactorial clinical condition leading to a rapid deterioration of the renal function associated with high morbidity, mortality (ranging from 25% to more than 50% depending on severity), and healthcare costs. In a large number of patients, AKI may be followed by irreversible and progressive chronic kidney damage (1–3).

Recent efforts have been made to standardize definitions and classification systems for AKI, and in 2004, the Acute Dialysis Quality Initiative first proposed the Risk, Injury, Failure, Loss, and End-Stage Renal Disease (RIFLE) criteria for diagnosis and classification of acute impairments in kidney function, which included five stages ranging from small changes in kidney function or urine output to kidney failure and end-stage renal disease (4). These criteria were subsequently refined into a three-stage system and further disseminated by the Acute Kidney Disease Network in 2007 (5, 6). In 2012, the KDIGO Clinical Practice Guideline for AKI consolidated these criteria into the most recent definition and classification system for AKI (7). The current definition and classification of AKI rely upon functional criteria including changes in serum creatinine (SCr) and urine output (6, 8–10). However, despite the harmonization in clinical definition and staging, identification of the complete biology and pathophysiology of AKI remains a major unmet need.

To this purpose, several research strategies have been undertaken to identify new cellular/biological elements implicated in the AKI-derived organ damage networking. Among them, the immune-inflammatory deregulation in this condition has been emphasized. As largely reported, the activation of immune-mediated mechanisms in AKI patients is a common thread, with immune cells playing a prominent role from initiating injury to promoting tissue repair (11, 12).

Renal epithelial cells, just after the acute offense, increase the expression of damage-associated molecular pattern (DAMP) molecules, Toll-like receptors (TLRs), and other alarmins (13–17) that, all together, facilitate a rapid recruitment to the site of injury of innate immune cells, including neutrophils, activated and resident macrophages, and dendritic cells (DCs) (18–21). Furthermore, in the last years, natural killer (NK) cells, extravasated from the vascular system to the site of injury, have been shown to play a role in the propagation of the immune response and the recruitment of adaptive immune cells (22–24).

In this review, we focus on NK cell populations found in the kidney, and we discuss their role in the induction and progression of AKI.

## Natural Killer Cells

NK cells are cytotoxic cells belonging to the broad family of innate lymphoid cells (ILCs) (25–28). Their effector functions range from the recognition and elimination of virus-infected and tumor cells to the secretion of cytokines/chemokines; importantly, they also display immune regulatory properties, exerted through interactions with different innate, and adaptive immune cells (29–31). In addition, they display the capability of sensing pathogens through TLR engagement and also to develop a kind of immunological memory (32–34). In order to exert these heterogeneous functions, NK cells use a large array of receptors able to sense stimuli from the microenvironment and, consequently, to mediate appropriate responses (35–37).

The recognition and elimination of abnormal cells can be fulfilled through receptor–ligand interactions involving several inhibitory and activating receptors and different types of ligands expressed on target cells. NK cells express multiple inhibitory surface receptors involved in the interaction with major histocompatibility complex (MHC) class I molecules and responsible for the “missing-self” recognition. The “missing-self hypothesis,” formulated by Ljunggren and Kärre (38) in the early 90s, postulated that NK cells can detect the absence of self MHC class I molecules on target cells. In humans, human leukocyte antigen (HLA) class I molecules expressed on autologous healthy cells allow the delivery of a negative signal, thus sparing normal cells from NK cell-mediated killing. On the other hand, virus-infected or tumor cells can lose or downregulate HLA class I expression, and the lack or dampening of the inhibitory interaction makes them susceptible to an NK cell-mediated attack. The main HLA class I-specific inhibitory NK cell receptors include killer immunoglobulin (Ig)-like receptors (KIR), leukocyte Ig-like receptor, subfamily B member 1 (LIR1)/Ig-like transcript 2 (ILT2), and the cluster of differentiation 94 (CD94)/NK group 2 member A (NKG2A) heterodimer. Collectively, they can recognize different HLA-A, -B, -C alleles, and non-classical HLA-E molecules, representing an efficient system to detect alterations in HLA class I expression (39–43). Later on, it was shown that recognition and killing of target cells by NK lymphocytes requires additional signals, mainly delivered by activating receptors (37, 44–47).

Besides CD16 (FcγRIII), representing the first characterized activating NK cell receptor and responsible for antibody-dependent cell-mediated cytotoxicity (ADCC), a variety of surface receptors and co-receptors, involved in the so-called natural cytotoxicity, were discovered over the years. The receptors playing a major role in the recognition of abnormal cells are represented by natural cytotoxicity receptors (NCRs, namely, NKp46, NKp30, and NKp44), NKG2D, and DNAX accessory molecule 1 (DNAM-1) (45, 48–55). While the ligands specific for NKG2D and DNAM-1 were identified long time ago and have been extensively characterized, ligands recognized by NCR started to be defined later, and the knowledge about NCR-ligand interactions is still incomplete (49, 56–61).

The best characterized ligands for activating NK receptors include molecules that are scarcely expressed on healthy/normal cells and that can be induced or upregulated following cellular stress, neoplastic transformation, and/or viral infection (52, 53, 55, 62–65). While many of these ligands are surface-expressed molecules, also nuclear proteins have been shown to bind to activating NK receptors following translocation to the target cell surface (66, 67). More recently, the landscape of NK cell receptor ligands has become even more heterogeneous in view of the finding that some NK cell receptors can also bind to secreted soluble factors, circulating molecules belonging to the complement system, or extracellular matrix components (68–70).

Given the multiplicity of receptor–ligand interactions, the engagement of NK cell receptors by specific ligands can result in opposite signals dictating the outcome of NK cell-mediated effector functions. NK cells can also respond to cytokines, including interleukin (IL)-12, IL-15, and IL-18 (mainly produced by myeloid cells upon inflammatory stimuli), and, in turn, release cytokines and chemokines, such as tumor necrosis factor (TNF)-α, interferon (IFN)-γ, granulocyte-macrophage colony-stimulating factor (GM-CSF), and CC-chemokine ligand 4 (CCL4) (30, 31, 71, 72).

Finally, the immunoregulatory role of NK cells has been deeply explored, starting from the characterization of NK–DC cross-talk (73–75). In this context, NK cells participate both in DC maturation and in the “DC editing” process through the recognition and killing of immature DCs that lack appropriate levels of MHC class I molecules. On the other hand, DC can favor NK cell proliferation and effector functions (76). More recently, the relevance of NK cells in immune regulation was further investigated, demonstrating the ability of NK cells to establish interactions also with other innate immune cells, i.e., macrophages and granulocytes, as well as with T lymphocytes (77–81).

## Heterogeneity of Natural Killer Cells

A further level of complexity arises from the heterogeneity of NK cells, i.e., the existence of different NK cell subsets characterized by distinct phenotypic and functional features and from their different localizations in the body (30, 72, 82, 83).

### Human Natural Killer Cells

In humans, NK cells were initially divided into two populations based on the expression of CD56 and CD16 surface markers (84). CD56^bright^CD16^dim/neg^ NK cells usually express the inhibitory HLA-E-specific receptor CD94/NKG2A but not KIR and low or undetectable CD16; they are poorly cytotoxic, being characterized by low intracellular levels of perforin and granzymes A and B, but can secrete high amounts of cytokines (primarily IFN-γ and TNF-α) in response to IL-2, IL-12, IL-15, and IL-18 (85, 86). According to their expression of chemokine and homing receptors (i.e., CCR7, CXCR3, CXCR4, and CD62-L), CD56^bright^ NK cells are mainly found in secondary lymphoid organs (SLOs), particularly in lymph nodes and tonsils, and also constitute a detectable fraction of NK cells in different organs and tissues (87). On the other hand, the CD56^dim^CD16^pos^ NK cell population is the predominant subset in peripheral blood, expresses NKG2A and/or KIR, and displays a high cytolytic potential and cytokine secretion capability following recognition of target cells expressing ligands for triggering NK receptors (88–90). Besides being more abundant in peripheral blood, the CD56^dim^ subset represents a remarkable fraction of NK cells found in spleen, bone marrow, and in certain non-lymphoid organs, such as lungs and kidney. CD56^dim^ NK cells can be further classified in different subsets based on distinct differentiation stages, the terminally differentiated one being represented by a KIR^pos^ CD57^pos^ CD16^bright^ subset which may express the activating HLA-E-specific receptor CD94/NKG2C (30, 91–96).

In addition, in recent years, it has also been discovered that, similar to adaptive T lymphocytes, also NK cells can undergo a process of clonal-like expansion and develop a kind of immunological memory. This concept was initially explored in the context of cytomegalovirus (CMV) infection, which was shown to modify the composition of the total NK cell repertoire and to drive a clonal-like expansion of given NK subsets (32, 97–100). In humans, these “memory” NK cells are distinguished by the expression of self HLA class I-binding KIRs, the terminal differentiation marker CD57, and the activating receptor complex CD94/NKG2C (101–103).

Finally, in the last decade, tissue-resident NK (trNK) cells were characterized as an additional NK cell population, resembling CD56^bright^ NK cells populating secondary lymphoid tissues but displaying markers of tissue residency/retention and mainly localized in non-lymphoid tissues (104–108). In view of these findings, the “traditional” CD56^dim^ and CD56^bright^ NK cell subsets, mediating a potent cellular cytotoxicity and able to produce IFN-γ, are often defined as conventional NK (cNK) cells.

The discovery of tissue residency markers, such as CD69, CD49a (α1 integrin), and CD103 (αE integrin), was essential for the characterization of these trNK cells. CD69, which for a long time has been considered an activation marker for T and NK cells, plays an important role in retaining cells in tissues, thus representing a marker of local residency, both in humans and in mice (109–111). In particular, CD69 inhibits sphingosine-1 phosphate receptor 1 (SIP1), specific for sphingosine-1 phosphate (SIP), which normally promotes the egress of lymphocytes from tissues into the blood. NK cells localized in different tissues have been shown to express CD69, while cNK cells derived from peripheral blood generally do not express this marker. CD103 and CD49a play a similar role in retaining cells in tissues, and their expression is induced by transforming growth factor-β (TGF-β) (112). Indeed, CD69, CD103, and CD49a markers allow to distinguish trNK cells from circulating cNK cells that are transiently recruited into tissues. Another possible mechanism related to tissue retention involves chemokines and chemokine receptors. While trCD56^bright^ NK cells found in lymphoid organs and liver are characterized by CXCR6 and CCR5 expression, circulating CD56^bright^ NK cells mainly express CCR7 (24, 107, 113).

### Murine Natural Killer Cells

In mice, NK cells are phenotypically characterized by the expression of several surface markers including CD161 (NK1.1), NKp46, the family of MHC class I-specific Ly49 receptors, and CD49b (α2 integrin, DX5) (35, 42, 114–118). While circulating cNK cells are defined as NKp46^+^ CD49a^−^ CD49b^+^, trNK cells display an NKp46^+^ CD49a^+^ CD49b^−^ phenotype (108, 119). Similar to human NK cells, cNK cell maturation in the mouse is a stepwise process, characterized by four stages according to CD11b and CD27 expressions (120, 121), with terminally mature NK cells being CD27^−^CD11b^+^ and also expressing KLRG1. Recent studies based on single-cell RNA-sequencing approaches have provided a more detailed view of murine NK cell developmental stages (122, 123). Five subsets have been identified on the basis of different genetic signatures, including the least mature NK and most mature NK clusters and three clusters defined as transitional NK subsets, which may represent intermediate steps of maturation or unique NK subsets that diverge late during development.

## Natural Killer Cells and Innate Lymphoid Cells

NK cells are not the only innate lymphocytes, being included in the family of innate lymphoid cells (ILCs) that are involved in homeostatic functions and in innate immune responses against different classes of pathogens (27, 28, 124, 125).

While cytokine release is a common feature of all ILCs, NK cells are the only cytotoxic cells among the ILCs. Initially, ILCs were divided into three main groups according to the expression of key transcription factors and distinct cytokine profiles. More recently, a greater heterogeneity of ILCs was appreciated, and these cells have been consequently classified into five subsets (NK cells, ILC1, ILC2, ILC3, and LTi cells) based on their development and function (27). NK cells share some features with ILC1, being characterized by the expression of T-bet transcription factor and the production of type I cytokines, such as IFN-γ and TNF-α. NK cells are the main ILCs found in peripheral blood, spleen, and bone marrow, whereas non-NK ILCs are more abundant in other secondary lymphoid tissues, including mucosa-associated lymphoid tissue (MALT) (126, 127). Notably, co-expression of T-bet and Eomesodermin (Eomes) transcription factors, besides cytotoxic potential, discriminates NK cells from ILC1. A further degree of complexity exists among ILC1 in relation to the heterogeneous expression of several markers. For instance, while ILC1 had been originally described as CD56^−^, the expression of CD56 can identify a subgroup of tonsil and intraepithelial ILC1 (ieILC1) characterized by cytotoxic granule expression and the ability to produce IFN-γ (125, 128, 129). In addition, CD56 can also be expressed by a subset of ILC3 (125, 130). ILC2 express GATA binding protein 3 (GATA3) transcription factor, display the ability to produce T helper type 2 (TH2)-like cytokines (i.e., IL-4, IL-5, and IL-13), and are tissue-resident. ILC3 are characterized by retinoic acid receptor-related orphan nuclear receptor gamma (ROR-γt) expression, produce TH17-like cytokines, and are abundant in the mucosae; based on the expression of NKp44 in humans (and of NKp46 in mice), they can be further divided into two subsets (131). LTi cells share with ILC3 the expression of ROR-γt transcription factor, but they have a distinct developmental path and are involved in the formation of secondary lymphoid structures (132). Since tissue residency is a general hallmark of ILCs, it has to be considered that some old studies analyzing NK cells in tissues should actually be reevaluated in view of recent findings concerning ILCs.

As for other organs, the presence of ILCs has been investigated in the kidney, revealing that group 2 ILCs represent the prevalent ILC population, both in mice and in humans, and can be expanded and activated by the epithelial cell-derived cytokines IL-25 or IL-33 (133–136). Notably, these cells can exert a protective effect in AKI through the induction of alternatively activated (M2) macrophages.

## Natural Killer Cells in the Kidney

The high heterogeneity of NK cells became more evident especially when their tissue localization was analyzed in different body compartments. It is now well-established that NK cells are found not only as circulating cells in peripheral blood, where they represent about 5–15% of lymphocytes, but also in SLOs as well as in different organs and tissues, in which specific NK cell subsets have been characterized (87, 105, 137).

NK cell trafficking from blood to tissues or lymphoid organs is coordinated by chemokines and their respective receptors, dictating the migration of different NK cell subsets to given compartments or to inflammatory sites (22–24). Notably, depending on the organ or tissue, NK cells can exhibit unique phenotypic characteristics and develop specific functional properties. trNK cells exhibit differences in terms of trafficking and tissue retention. Interestingly, trNK cells residing in different districts share some common features but also peculiar properties that might reflect the influence of the local microenvironment in shaping these cells (104–106, 108, 119). The body districts with the highest prevalence of NK cells are the liver (137–144), lungs (145–147), and uterus (148–151). NK cells have also been found in several other organs including the kidney, intestinal mucosa, breast tissue, synovia, pleural and peritoneal fluids, skin, salivary glands, and adipose tissue (105, 137, 152–154). Notably, the relative distribution of CD56^bright^ and CD56^dim^ NK cell subsets is heterogeneous in different tissues; while in most cases, CD56^bright^perforin^low^ cells (non-cytotoxic) represent the prevalent subset, the lungs contain a higher proportion of CD56^dim^ perforin^high^ NK cells, and the kidney is populated by intermediate levels of these two NK cell populations.

NK cells represent about 25% of lymphocytes in the healthy human kidney, with an enrichment in the CD56^bright^ NK cell subset as compared with peripheral blood (137, 155–157). Until recently, however, information on tissue-resident lymphocyte populations, and in particular on trNK cells in the kidney, had been relatively limited. Although the presence of both innate and adaptive lymphocytes in this district was known for a long time, it was not clear whether these cells displayed features of tissue residency, similar to what was previously observed in other organs and tissues. In recent years, several studies helped to clarify this issue both in mice and in humans. In this context, parabiosis experiments performed in mouse models proved to be very effective in demonstrating the presence of trNK cells in the kidney.

The study by Victorino et al. in the mouse showed that about 15–20% of NK cells in the kidney are represented by a tissue-resident CD49a^+^ DX5^−^ NK cell population reminiscent of trNK cells harbored in other organs, such as the liver and uterus (158). The discrimination between trNK cells and ILC1 residing in non-lymphoid tissues is crucial, in view of the similarities between these two cell populations, including the CD49a^+^DX5^−^ phenotype. Studies performed in different tissues allowed to establish that most murine ILC1 are CD127^+^ (IL-7Rα^+^) and do not express the Eomes transcription factor, while murine trNK cells are CD127^−^ and depend on Eomes for their development. In addition, CD200R1 surface marker has been associated with ILC1 but not with NK cells (27, 127, 159, 160). Finally, trNK cells display some cytotoxic capability thanks to the expression, albeit at low/moderate levels, of perforin and granzymes, whereas ILC1 (except for ieILC1) are non-cytotoxic cells. Although not all these markers have been analyzed so far in renal NK cells, it is conceivable that trNK cells in the kidney may share several markers with trNK cells that have been better characterized in other tissues.

The murine kidney also harbors a substantial number of CD49a^−^ DX5^+^ NK cells that are considered cNK cells passing through the organ. Contrary to cNK cells, kidney trNK cells do not require NFIL3 and Tbet transcription factors for their development and express lower levels of Asialo-GM1 (AsGM1) as compared to CD49a^−^DX5^+^ cNK cells; this finding suggests that NK cell depletion by anti-AsGM1 antibodies could be incomplete/inefficient and gives the possibility to investigate trNK cell function in animal models. Indeed, selective (by anti-AsGM1 mAb) or total (by anti-NK1.1 mAb) depletion of NK cells allowed to assess the predominant role of trNK cells in a model of ischemic AKI (see below).

In addition, NK cells residing in the kidney are very efficient in producing IFN-γ, and this property has been shown to play an important role in progressive tubule-interstitial fibrosis and chronic kidney disease (CKD) (155). IFN-γ can induce the production of profibrotic factors, such as transglutaminase 2 (TG2) and the heparan sulfate proteoglycan syndecan-4 (sdc4) that contribute to the accumulation of extracellular matrix, thus favoring the development of renal fibrosis. This issue has been recently explored in a murine model of aristolochic acid nephropathy (AAN), in which the presence of trNK cells positively correlated with the progression of tubule-interstitial fibrosis (161).

Also in humans, healthy kidney harbors a relevant NK cell compartment: NK cells represent approximately 25% of total lymphocytes; both CD56^bright^ and CD56^dim^ NK cells can be found, with a higher proportion of CD56^bright^ NK cells in the kidney (about 37% of total NK cells) as compared to peripheral blood (>10%) (137). Interestingly, a recent study, analyzing kidney biopsies from patients with different renal diseases, revealed the existence of a CD56^bright^ NK cell population with tissue residency features (CD69 expression) and the ability to release IFN-γ (155).

## Natural Killer Cells in Acute Kidney Injury

AKI is a clinical condition characterized by acute impairment of kidney function and induced by different causes, including ischemia, sepsis, and toxic insults (1, 162–164). In particular, ischemia–reperfusion injury (IRI) is one of the most frequent events leading to severe AKI.

A common hallmark of severe AKI is the occurrence of acute tubular necrosis. In the kidney, different parenchymal cells, including tubular epithelial cells (TECs) and endothelial cells, can respond to DAMPs or to pathogen-associated molecular patterns (PAMPs) through several TLRs and/or inflammasome components and thus contribute to renal inflammation. Indeed, several DAMPs, released as a consequence of tissue damage, or PAMPs expressed by infectious agents, can activate not only innate immune cells but also non-immune cells. Several studies concerning the role of TECs in kidney injury confirmed an active role for these cells both in the induction and in the regulation of inflammatory responses (13–17, 165, 166).

The expression of TLR2 and TLR4 on TECs allows these cells to sense endogenous inducers of inflammation and subsequently to be activated to produce several cytokines and chemokines (167). In particular, TLR2 involvement has been assessed in kidney IRI, where hypoxic conditions can induce tubular necrosis and the consequent release of endogenous TLR ligands, which will act at both autocrine and paracrine levels (i.e., on endothelial cells and on innate immune cells) (13, 16, 168, 169). DAMPs are also recognized by renal DCs, which contribute to the inflammatory response and to neutrophil recruitment by the secretion of inflammatory mediators, cytokines, and chemokines.

### Tubular Epithelial Cell–Natural Killer Cell Interactions

**Figure 1:**
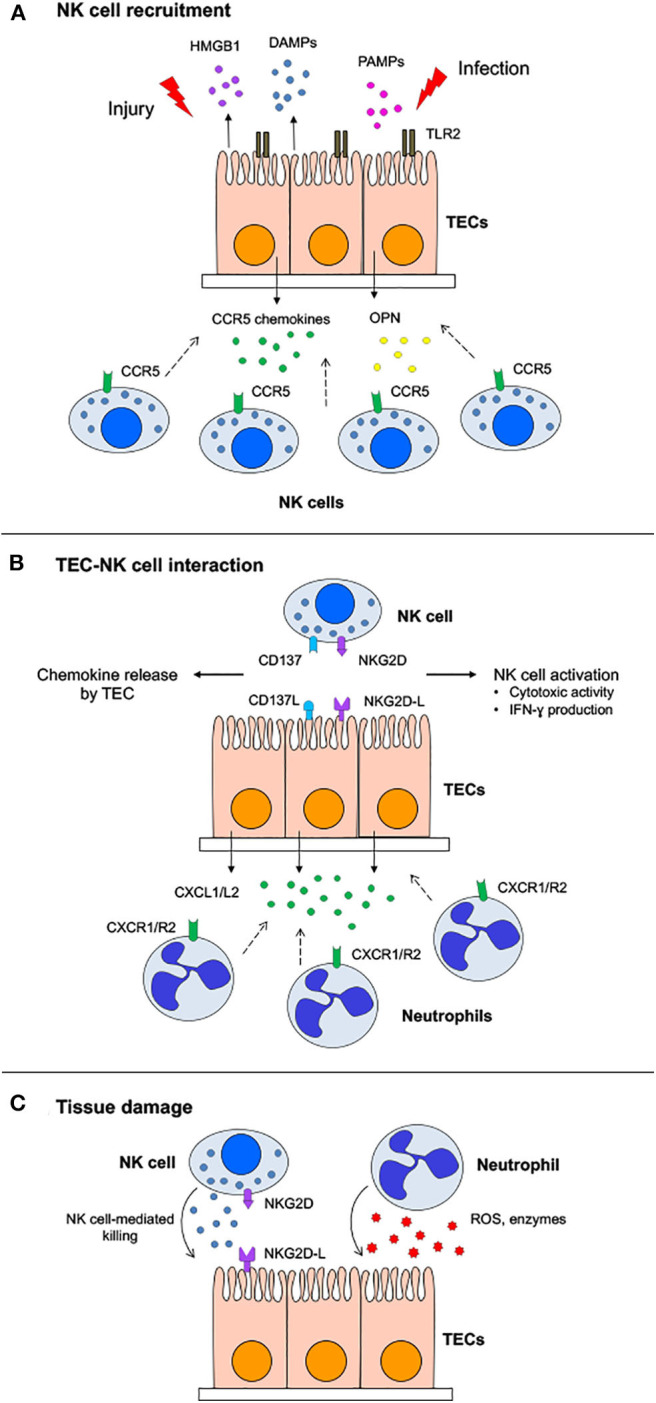
Role of natural killer (NK) cells in acute kidney injury. **(A)** Following acute kidney injury, damage-associated molecular patterns (DAMPs) released by damaged tubular epithelial cells (TECs) or pathogen-associated molecular patterns (PAMPs) derived from infectious agents are recognized by pattern recognition receptors expressed on TECs that in turn release osteopontin (OPN) and CCR5 chemokines able to recruit NK cells. **(B)** TEC–NK cell cross-talk occurs through different receptor–ligand pairs. NKG2D ligands (NKG2D-L), upregulated on TECs, engage NKG2D on NK cells, inducing both cytotoxic activity and interferon (IFN)-γ production. On the other hand, CD137–CD137L interaction stimulates in TECs the secretion of chemokines attracting neutrophils. **(C)** TECs are killed by NK cells through the release of cytotoxic granules, while activated neutrophils are responsible for tissue damage due to reactive oxygen species (ROS) and lytic enzymes.

Of particular interest is the interplay occurring between NK cells and TECs in the context of kidney injury, especially in view of the finding that NK cells are recruited in the earliest stages of IRI, already 4 h after injury (Figure 1) (170). The injured TECs release high-mobility group box protein 1 (HMGB1), an endogenous TLR2 ligand released following tissue damage that stimulates CCR5 chemokine production through TLR2 in an autocrine manner. CCR5 chemokines (CCL3, CCL4, and CCL5) in turn mediate recruitment of NK cells that induce TEC to release CXCL1 and CXCL2 chemokines, responsible for the accumulation of neutrophils in the kidney (169). Overall, TECs play a critical role in the induction and orchestration of acute renal inflammation by regulating the sequential migration of NK cells and neutrophils into the kidney during the early phase of IRI.

The involvement of NK cells in IRI was further supported by the finding that, in mice, the expression of ligands for the activating receptor NKG2D (Rae-1, MULT-1, and H60), is increased during kidney IRI and is paralleled by a concomitant rapid NK cell infiltration in injured kidney (171–173). This seems mediated by HMGB1 through engagement of TLR4 and subsequent MyD88-dependent signaling (174). The role of TLR4 was further confirmed by *in vitro* experiments showing RAE-1 and MULT-1 upregulation on isolated TECs following lipopolysaccharide (LPS) exposure (173).

Both in murine and human TECs, the expression of ligands specific for activating NK cell receptors has been demonstrated, suggesting that these receptor–ligand interactions could be involved in the recognition and killing of TECs. Thus, activated spleen-derived murine NK cells were shown to efficiently kill syngeneic TECs *in vitro* mainly through the engagement of NKG2D activating receptor by Rae-1 ligand expressed on TECs and by the use of perforin (172).

Similarly to what was observed in murine models, human NK cells display the *in vitro* ability to kill TECs (HK-2 cell line) exposed to hypoxia, a condition mimicking ischemic AKI, following the interaction of NKG2D receptors with MHC class I chain-related protein A (MICA), whose expression is upregulated in human TECs by hypoxia-inducible factor-1 alpha (HIF-1α) transcription factor (175). One possible mechanism of MICA upregulation in hypoxic conditions involves TGF-β, a cytokine playing multifunctional roles in inflammation, injury, and tissue repair and induced in the kidney and in TECs, following ischemic injury (176, 177). It is of note, however, that TGF-β expression has been shown to correlate with limitation of renal IRI, better TEC survival, and protection against NK cell-mediated killing (177, 178).

These effects can be explained by the fact that TGF-β, besides increasing MICA surface expression on TECs, also induces higher levels of soluble MICA, a well-known mechanism of modulation of NK cell-mediated cytotoxic activity (62, 179). In addition, TGF-β exerts a regulatory role on NK cell function mainly through the downregulation of different activating receptors, including NKG2D and NKp30 (180, 181). In view of these findings, the modulation of surface and soluble MICA expression could represent a useful strategy to reduce renal injury.

Although the mechanisms responsible for NK cell recruitment and activation in renal IRI have not been fully elucidated, a role for osteopontin (OPN) has been demonstrated. OPN is a secreted glycoprotein expressed in different immune cells, including NK cells, and exerting pro-inflammatory functions (182–184). Notably, mRNA and protein OPN expression is increased in the kidney shortly after IRI (185–187), and OPN has been shown to promote ischemic kidney injury (186, 187).

The role of OPN, however, is still debated since a protective effect for OPN both in kidney IRI and in tissue repair was reported (188). Interestingly, it has been shown that TECs display the ability to secrete high levels of OPN, which in turn can induce a rapid NK cell migration with an indirect, still undefined, mechanism, possibly involving the induction of chemokines or other chemotactic factors able to recruit NK cells. In addition, OPN can activate NK cells and increase their cytotoxic activity against primary TECs (187).

More recently, the involvement of OPN in renal injury following ischemia–reperfusion was further validated by Cen et al. in an *in vivo* model. This study confirmed an OPN increase following IRI, both at the mRNA and protein levels, and demonstrated that neutralization of OPN by an anti-OPN mAb resulted in a decreased NK cell infiltration in the kidney associated with a reduced severity of renal injury, lower levels of pro-inflammatory cytokines, and decreased neutrophil infiltration (189). Interestingly, high OPN expression was also observed in kidney grafts, and chronic transplant kidney injury was abrogated in OPN-deficient kidney grafts after transplantation, suggesting that OPN could play a role also in kidney allograft injury (190).

The search for additional TEC–NK cell interactions involved in renal IRI led to the characterization of the co-stimulatory CD137–CD137 ligand (CD137L) axis. Previously, several reports had already pointed to a role for CD137–CD137L interaction in inflammation. CD137L expressed on professional APC can co-stimulate TH1 helper T cells through the engagement of CD137; on the other hand, reverse signaling induced on APC can promote cytokine and chemokine secretion.

In the context of renal IRI, CD137 expression on activated NK cells results in the transmission of a “reverse signal” on TECs through the binding to CD137L; in turn, TECs produce high levels of CXC chemokines, such as CXCL1 and CXCL2, responsible for neutrophil recruitment and the subsequent acute inflammatory response (191).

Indeed, in a mouse model of acute IRI, the expression of CD137 on NK cells and CD137L on TECs was required for kidney injury. In addition, NK cell depletion experiments demonstrated the essential role of NK cells in neutrophil recruitment and the resulting renal injury. Depletion of neutrophils abrogated renal IRI as well, suggesting that neutrophils were directly responsible for tissue damage associated with renal IRI, while NK cells were responsible for neutrophil recruitment. In this context, it is of note that NK cells can be rapidly recruited in the kidney, within 4 h after IRI, and upregulate CD137 surface expression, suggesting their important role in the first phases of acute tissue damage.

The role of trNK cells as central mediators of ischemic tissue injury was clearly demonstrated in a model of ischemic AKI (158). The analysis of both cNK and trNK cells at 4 and 24 h after reperfusion revealed that IRI did not modify either the relative distribution or the original phenotype of these two cell subsets. Notably, trNK cells were characterized by a higher expression of several markers, including CD160, CD44, and TRAIL, suggestive of a higher activation state, and by lower levels of KLRG1 and CD244 inhibitory receptors. Based on their tissue residency and activation state, trNK cells can exert a prominent role in the early local response during IRI; it is conceivable that cNK cells recruited into the tissue can further enhance tissue damage.

IRI is an inevitable event associated with kidney transplantation. Being actively involved in the induction of inflammatory responses, TECs play a major role in this process (13, 17, 168, 192). Moreover, in kidney transplant rejection, TECs represent one of the major targets of the alloreactive immune response mediated by CD8^+^ T lymphocytes and NK cells. The study by Demmers et al. analyzed the *in vitro* susceptibility of primary donor-derived TECs activated by IFN-γ and TNF-α to CTL- and NK cell-mediated killing. While unstimulated allogeneic TECs were efficiently killed by both CD8^+^ T cells and NK cells of the recipient, cytokine-activated TECs became more resistant to NK cell-mediated killing presumably because of the increased expression levels of HLA class I molecules. This study also evaluated the effect of different immunosuppressive drugs on immune-mediated TEC lysis, showing their limited efficacy *in vitro* and differential inhibitory effects on CTL vs. NK cells (193).

## Concluding Remarks

In recent years, the knowledge about blood-derived and tissue-resident NK cells found in the kidney is improved, revealing once again the complexity and the versatility of this ILC population (Table 1). In this context, the role of NK cells has also been addressed in immune-mediated pathologic conditions affecting the kidney. For instance, it is now clear that NK cells are involved in the pathogenesis of AKI, as demonstrated both in animal models and in humans. In particular, the role of NK cells in AKI can occur by distinct mechanisms, including (i) NK cell recruitment and activation mediated by CCR5 chemokines (directly) or OPN (indirectly) secreted by TECs; (ii) secretion of neutrophil-attracting chemokines induced in TECs through the CD137–CD137L axis; iii) NK cell-mediated killing of TECs through NKG2D–NKG2D-L interactions.

**Table 1:** NK cell populations described in human and murine kidney.

**Human NK cells**		
**Phenotype**	**Main observations**	**References**
CD45^pos^CD3^neg^CD94^pos^CD56^dim^ perforin^high^ CD45^pos^CD3^neg^CD94^pos^CD56^bright^ perforin^low^	NK cells: 25% of total lymphocytes in the kidney CD56^bright^ subset: 37% of total NK cells	(137)
CD3^neg^CD56^dim^CD16^pos^ CD3^neg^CD56^bright^CD16^neg/low^CD69^pos^	CD56^bright^ subset involved in tubulointerstitial fibrosis CD56^bright^ subset: IFN-γ production	(155)
**Murine NK cells**		
**Phenotype**	**Main observations**	**References**
CD45^pos^CD3^neg^DX5^pos^	NK cells involved in kidney IRI	(172, 187, 189)
CD45^pos^CD3^neg^NK1.1^pos^	NK cells involved in kidney IRI	(191)
NK1.1^pos^NKp46^pos^CD49a^pos^DX5^neg^AsGM1^low^ (trNK) NK1.1^pos^NKp46^pos^CD49a^neg^DX5^pos^AsGM1^high^ (cNK)	cNK and trNK cells described in the kidney trNK cells involved in kidney IRI	(158)
CD3^neg^NKp46^pos^DX5^neg^ (trNK) CD3^neg^NKp46^pos^DX5^pos^ (cNK)	trNK cells involved in tubulointerstitial fibrosis trNK cells: accumulation in fibrotic tissue and IFN-γ production	(161)

*NK, natural killer; IRI, ischemia–reperfusion injury; cNK, conventional NK cells; trNK, tissue-resident NK cells; AsGM1, Asialo-GM1; IFN-γ, interferon-γ*.

Therefore, because of their major involvement in AKI pathogenesis, NK cells could represent a novel target for future strategies for this important clinical condition.

## Author Contributions

CC and GZ wrote the manuscript. SG, MB, GS, and GC reviewed the manuscript and provided critical input. All authors listed have made a substantial, direct and intellectual contribution to the work, and approved it for publication.

## Conflict of Interest

The authors declare that the research was conducted in the absence of any commercial or financial relationships that could be construed as a potential conflict of interest.
